# Stratified support pattern-based internet-assisted self-management therapy for diabetes mellitus -mild cognitive impairment: a randomized controlled trial protocol

**DOI:** 10.1186/s12902-023-01485-1

**Published:** 2023-11-02

**Authors:** Yun-xian Wang, Ji-xing Liang, Rong Lin, Yuan-jiao Yan, Hong Li, Ming-feng Chen

**Affiliations:** 1 The School of Nursing, Fujian Medical University, No. 88 Jiaotong Road, Fuzhou City, 350004 Fujian Province China; 2 Nursing Department, The First People’s Hospital of Yunnan Province, No 157 Jinbi Road, Kunming City, 650032 Yunnan Province China; 3 Endocrinology Department, Fujian Provincial Hospital & Shengli Clinical Medical College, No. 134 East Street, Fuzhou City, 350122 Fujian Province China; 4 The School of Nursing, Fujian Medical University, No. 1 Xuefu North Road, Fuzhou City, 350122 Fujian Province China; 5 Research Center for Nursing Theory and Practice, Fujian Provincial Hospital & Shengli Clinical Medical College, No. 134 East Street, Fuzhou City, 350122 Fujian Province China; 6 Neurology Department, Fujian Provincial Hospital & Shengli Clinical Medical College, No. 134 East Street, Fuzhou City, 350122 Fujian Province China

**Keywords:** Mild cognitive impairment, Diabetes mellitus, Self-management, Stratified support pattern-based, Internet-assisted

## Abstract

**Background:**

Mild cognitive impairment (MCI) associated with diabetes mellitus (DM) is common among older adults, and self-management is critical to controlling disease progression. However, both MCI and DM are heterogeneous diseases, and existing integrated self-management interventions do not consider patient differences. Grouping patients by disease characteristics could help to individualize disease management and improve the use of available resources. The current study sought to explore the feasibility and effectiveness of a stratified support model for DM-MCI patients.

**Methods:**

Eighty-four DM-MCI patients will be randomly divided into an intervention group and a control group in a 1:1 ratio. The intervention group will receive a self-management intervention using the stratified support pattern-based internet-assisted therapy (SISMT), while the control group will receive the health manual intervention (HMI). The study recruiter will be blinded to the group allocation and unable to foresee which group the next participant will be assigned to. At the same time, the allocation will be also hidden from the research evaluators and participants. After 12 weeks and 24 weeks, cognitive function, blood glucose, self-management ability, psychological status, health literacy, and self-management behavior of patients in both groups will be measured and compared.

**Discussion:**

This study developed a stratified support pattern-based internet-assisted to provide self-management intervention for patients with DM-MCI. The impact of different models and forms of self-management intervention on cognitive function, blood glucose management, and psychological status health literacy and self-management behavior of patients will be assessed. The results of this study will inform related intervention research on the stratified support pattern-based internet-assisted self-management therapy, and help to slow the decline of cognitive function in patients with DM-MCI.

**Trial registration:**

ChiCTR2200061991. Registered 16 July 2022.

## Background

Diabetes mellitus (DM) is one of the chronic diseases that develop rapidly and threaten global health. According to the data of the International Diabetes Federation (IDF) in 2021, about 537 million adults (20–79 years old) worldwide suffer from diabetes, which will rise to 783 million by 2045. During this period, the world population is estimated to increase by 20%, while the number of patients with diabetes is estimated to increase by 46% [1]. It can be seen that the form of diabetes is not optimistic. DM is characterized by persistent hyperglycemia, which will not only cause chronic damage to blood vessels, brain and other tissues and organs [2, 3], but also affect the nervous system function of patients, thus causing cognitive impairment [4]. Some studies have further proved the correlation between this disease and found that most diabetes patients will have cognitive impairment, and 25–36% of them have mild cognitive impairment (MCI) [5]. Some research teams have called this type of patient as DM-MCI [6–8], and one of research teams conducted a survey on the prevalence of this type of patient. It was found that the overall prevalence of DM-MCI is 45%, indicating that DM-MCI is affecting residents on a large scale [8].

MCI refers to a stage in which a patient's cognitive function has been impaired, but its severity is not sufficient to constitute dementia, and it is a precursor to the development of dementia [9]. According to statistics, the global prevalence of elderly MCI is approximately 3–22%, with approximately 5–10% of these patients developing dementia annually [10, 11]. As one of the risk factors for cognitive impairment, diabetes not only increases the risk of cognitive impairment, but also accelerates the disease progression of MCI. Fortunately, mild cognitive function has a characteristic of bidirectional transformation. It means that MCI is an intervention stage in disease progression [12]. And a study has found that MCI patients' self-management ability related to blood glucose control is poor, which will affect the blood glucose control of patients. This undoubtedly further accelerated the progress of cognitive impairment in patients with DM-MCI [13]. Therefore, improving self-management ability has become an important measure to prevent the deterioration of disease in patients with DM-MCI.

Self-management is a dynamic and continuous self-regulation process that utilizes patients' own skills and knowledge input to effectively control their life confidence and motivation, leading to behavioral changes. It is an important method for controlling and slowing down the progression of chronic diseases, thereby affecting patients' psychological state, quality of life, and happiness [14]. While strong self-management can delay disease deterioration, poor self-management can increase disease progression [15]. Prior studies on the self-management of patients with DM and/or MCI have achieved positive results. In a study by Katja et al., patients with type 2 diabetes used telemedicine-assisted self-management behavior over a 3-month period [16]. The intervention included diet, physical activity, blood glucose monitoring, first aid training, and clinical and stress management, and telephone guidance was conducted based on each patient’s personal needs. Glycated hemoglobin levels and self-management behavior scores improved significantly in response to the intervention. Yang Q et al. developed self-management measures based on a forgetting pattern of the human brain towards new things to provide intervention to patients with MCI [17]. After 3 months, there were improvements in the cognitive function and daily living ability of the patients. However, the intervention for DM-MCI patients are mostly single content, such as cognitive training or diet only for patients [18, 19]. Few studies have focused on comprehensive self-management interventions that are individualized and do not use an integrated intervention mode.

MCI and DM are heterogeneous diseases, and this heterogeneity increases when the conditions co-occur in the same individual [20, 21]. Personalized intervention is an important way to address this issue [22]. However, in populous countries like China [23], it can be difficult to implement targeted intervention measures. In addition, personalized medicine requires many human resources, making it a challenge to sustain it over a long time period. To address this, relatively individualized, or “stratified” intervention measures, were developed. The meaning of “stratified” intervention is to classify patients based on their characteristics of diseases, and provide corresponding intervention for various categories of patients. Many studies have adopted such “stratified” intervention. Li et al. used applied acute physiology and chronic health evaluation II (APACHE II) to evaluate the illness severity and self-care ability of hemodialysis patients and divided the patients into three categories for stratified intervention [24]. Patients in each category were given an aerobic resistance exercise routine with the same content but a different frequency. Hill et al. and other research teams classified patients with low back pain into low, medium, and high-risk categories to enable stratified management. The stratified pattern greatly aided disease management [25]. It is important to note that these programs were designed to target a single disease. Different methods have been used to give patients the same intervention content at a different frequency, saving human resources and improving disease control. Thus, it is of great significance to explore the impact of stratified self-management on the outcomes of patients with DM-MCI.

Routine self-management often includes the use of offline educational materials such as health manuals and lectures. However, the Internet is not limited by space and time, which makes it an important tool for training and education. “Internet-assisted” medical treatment is being increasingly applied in nursing [26]. During the COVID-19 pandemic, this intervention has been used to optimize medical structures, dynamically monitor changes in patient conditions, and provide guidance for rapid rehabilitation [27]. Studies indicate that this has worked to effectively reduce the risk of viral spread and improve the efficiency of medical treatment [28]. Among numerous internet-assisted interventions, WeChat has become a commonly used social media software for Chinese researchers and patients, and many studies have adopted WeChat as a medium for internet-assisted self-management interventions, achieving positive results [29, 30]. It is worthwhile to evaluate the use of a stratified support pattern-based internet-assisted self-management therapy for DM-MCI with WeChat as the intervention medium. And randomized controlled trial (RCT), as an important method widely used in the clinical verification of intervention technology around the world, has been applied to the self-management research of more patients with mild cognitive impairment or diabetes patients [31]. Therefore, we will use RCT as the research design method to verify the effectiveness of stratified support pattern-based internet-assisted self-management therapy(SISMT) for DM-MCI.

The current study has designed a self-management intervention using the “Internet-assisted” stratified support-pattern may be able to consider the heterogeneity of patients more and provide them with more personalized intervention strategies. The aim is to use this approach to address the heterogeneous needs of patients with DM-MCI. Stratification was determined based on the cluster classification of patient cognitive function and self-management ability. Using these characteristics along with expert guidelines and consensus [32, 33], intervention measures will be implemented for a duration of 12 weeks. The effectiveness of the intervention on the self-management and cognitive function of each group of patients with DM-MCI will be explored.

### Study objectives and hypotheses

The primary objective of the study is to compare the effectiveness of a 12-week stratified support pattern-based internet-assisted self-management therapy(SISMT) and a health manual intervention mode(HMI) on the cognitive function of DM-MCI patients. The secondary objectives are to evaluate the effectiveness of the interventions on fasting blood glucose (FBG), HbA1c, Postprandial blood glucose (PBS) levels, depression, anxiety, quality of life, happiness, self-management ability, and diabetes health literacy.

The first primary hypothesis is that the cognitive function scores will show more improvement among participants in the SISMT group than those in the HMI group 12 weeks after the intervention. The secondary hypotheses are that participants in the SISMT group will have (1) better blood glucose control (FBS, HbA1c, PBS). (2) lower depression and anxiety scores, (3) higher quality of life and happiness scores, (4) improved self-management, and (5) better health literacy than those in the HMI group 12 weeks after the intervention.

### Theoretical framework

**Fig. 1:**
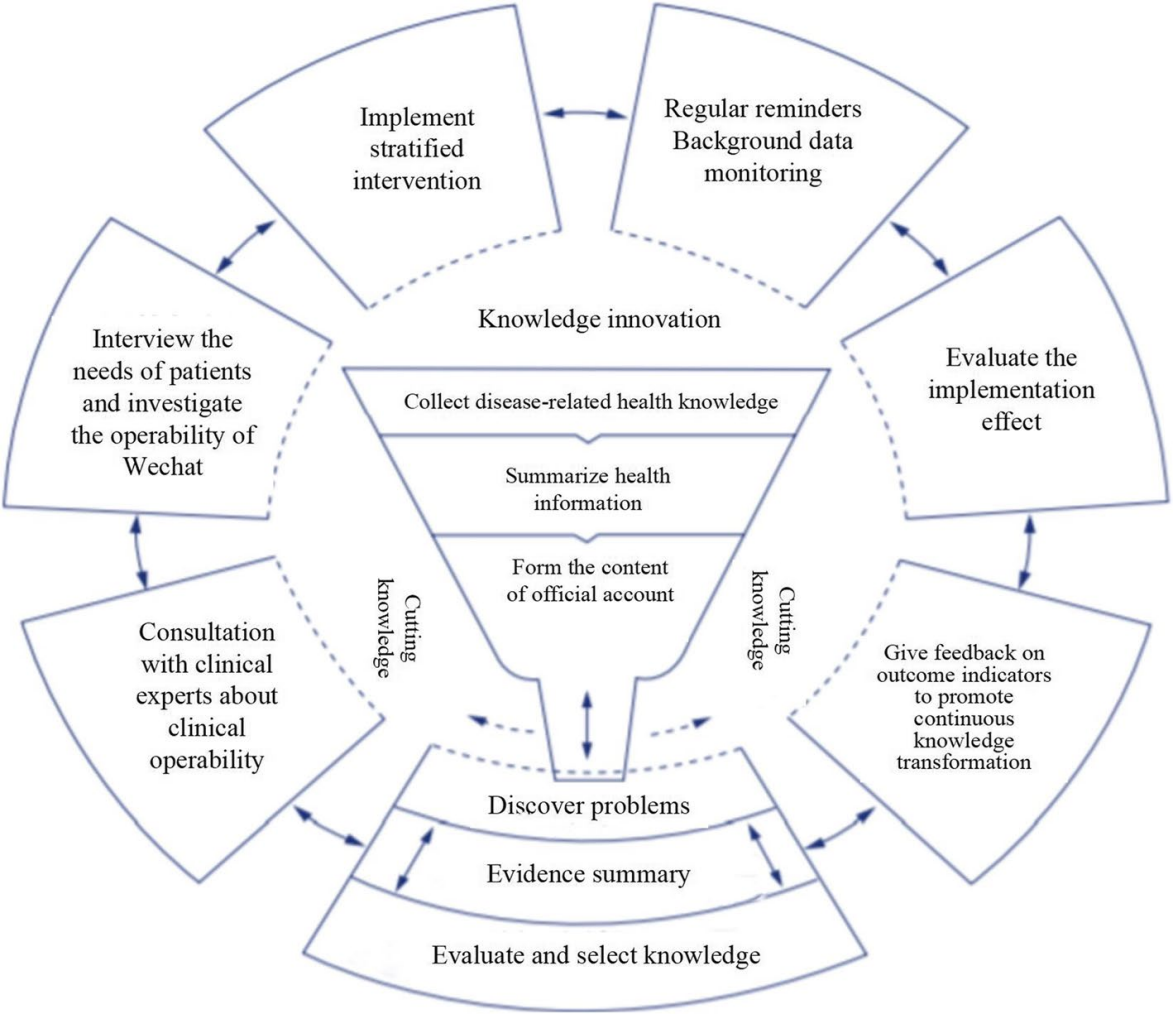
Theoretical framework of the study: the Knowledge to Action Cycle (KTA)

This study is based on the Knowledge to Action Cycle (KTA) framework developed by Graham et al. [34]. According to the KTA framework, knowledge to practice is a cyclic, dynamic, and complex process in which knowledge innovation and application are interlinked. The KTA includes seven steps, researchers participate in the entire research process, which this study will combine as follows: 1) Find problems and extract relevant knowledge to self-manage DM-MCI according to expert guidelines and consensus; 2) Consult with clinical experts about the operability of the intervention; 3) Assess patient needs and investigate the operability of using Wechat for older adults; 4) Implement the self-management stratified model in patients with DM-MCI; 5) Establish different reminder frequencies based on patient characteristics and review Wechat background data; 6) Measure outcome indicators and evaluate the intervention; 7) Provide feedback to the patients and urge them to continue the knowledge behavior transformation (Fig. 1).

## Methods

### Trial design

This is a single-blind, two-arm randomized controlled trial with randomization at the participant level. Participants will be randomly allocated to receive a HMI (control) or a SISMT (intervention). Primary and secondary study outcomes will be collected for both groups at baseline (T0) and 12 weeks(T1), 24 weeks post-intervention(T2). As the study involves the older adults with MCIs, a few specific ethical procedures should be followed and ensured. Only those who signed the consent form can be included in this study, and a guardian as the legal representative should be asked to co-sign the consent form. The trial has been registered at ChiCTR.org.cn (ChiCTR2200061991, date:16/07/2022).

### Study aim, setting and ethics

**Fig. 2:**
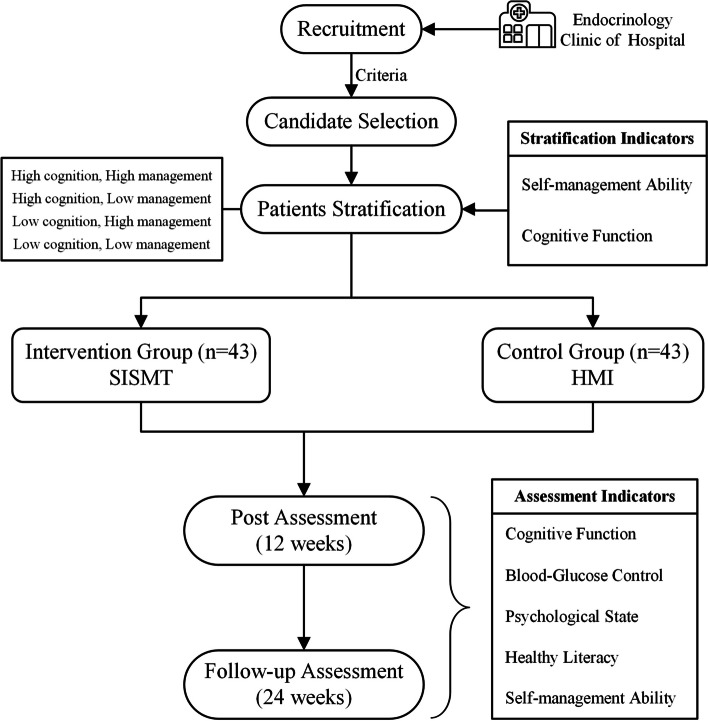
Flowchart of the SISMT for DM-MCI

To evaluate the effectiveness of a SISMT for DM-MCI will be conducted, with allocation concealment and outcome assessor blinding (Fig. 2).

### Participants

#### Recruitment and screening

By convenient sampling, patients with DM will be screened for MCI and included in the study. This study is approved by relevant hospital departments and most patients were recruited from the endocrine clinic at a provincial Third-Grade Class-A General Hospital. The study researchers will met with DM patients who were visiting the clinic and asked them to complete the Montreal Cognitive Assessment (MoCA) [35], the Mini-Mental State Examination (MMSE) [36], the diabetes self-management scale (DSCS) [37], and the Activity of Daily Living (ADL) survey [38], along with a general information questionnaire. The questionnaires will be completed in separate rooms of the hospital. So far, 240 DM-MCI patients have been screened.

#### Diagnosis criteria

MCI will be diagnosed using Petersen diagnostic criteria as follows [39]: 1) Chief complaint: memory decline; 2) MoCA score of 13–14 for illiteracy, 19–20 for patients with primary school education(1–6 years education), 24–25 for those with junior high school education(≥ 7 years education) [40]; 3) An MMSE score of 24–30 indicates the absence of dementia [41]. (4) ADL will be used to measure the intact activities of daily living (Lawton–Brody ADL score < 18) [38].

#### Inclusion criteria

1) Meet the diagnostic criteria for diabetes (The diagnosis of diabetes is based on the doctor's diagnosis results in the hospital diagnosis and treatment system). 2) Meet the diagnostic criteria for mild cognitive impairment. 3) ≥ 60 years of age. 4) No obvious visual or hearing impairments. 5) Participants have good communication skills and can cooperate with researchers to complete the survey questionnaire. 6) Able to use Internet device dependently.

#### Exclusion criteria

1) Serious physical diseases that hinder completion of the cognitive function screening. Such as severe Parkinson, paralysis and so on. 2) Drug or alcohol dependence. 3) Other nervous system diseases and/or serious medical conditions that can alter brain function. 4) Inability to use smartphones independently.

### Sample size calculation

The required sample size is estimated using PASS v11.0 (NCSS, Kaysville, UT, USA) based on a completely random design for comparing the means of two independent samples. To our knowledge, there is no previous study on a stratified support pattern-based internet-assisted self-management therapy for DM-MCI in mainland China, so we estimate the effect size of one of the outcomes (HbA1c) based on previous research on patients with DM-MCI [42]. This study find that HbA1c scores in intervention and control group are 6.49 ± 1.59 and 6.97 ± 0.73, respectively. A sample size of 34 participants per group was determined to be sufficient to detect an effect with a type 1 error rate of 5% (α = 0.05) and 90% power (β = 0.1). A total of 68 participants will be needed, with 34 participants per group. Considering a 20% loss of follow-up rate, the final sample size is 86, with 43 participants per group.

### Randomization, blinding, and concealed allocation

Ensuring allocation concealment, participants will be randomized (after obtaining written informed consent, eligibility screening, and baseline assessment) to the intervention and control groups at a 1:1 ratio by a study staff member (who will not be involved in participant recruitment or outcome assessment) using Research Randomizer software (http://www.randomizer.org/). Participants in the intervention group will be assigned to different categories of the intervention group based on their cognitive function and self-management characteristics and will receive Intervention measures with different frequencies. Participants in the control group will be assigned to different categories of the control group based on their cognitive function and self-management characteristics, too, but there is no difference in intervention frequency. The participants will then be told their group assignment by the intervention staff. Due to the nature of non-pharmacological interventions, only the outcome assessors and data analysts (not the participants or intervention staff) will be blinded to group allocation.

### Intervention

#### Intervention group

In this study, the intervention group will adopt a SISMT. After the completion of the baseline survey, participants will formulate personalized intervention measures and optimize and expand their knowledge through hierarchical strategies to improve their self-management behavior. Based on our previous research in this study, the patients will be divided into four categories according to cognitive function and self-management ability: (1) high cognitive high management, (2) high cognitive low management, (3) low cognitive high management, and (4) low cognitive low management. Four WeChat groups will be formed to intervene separately to prevent communications between each category of participants in the intervention group. Self-management interventions targeted to patients with DM-MCI will be implemented, including diet, exercise, drug intake, psychology, and cognition. To achieve stratified intervention with different intensity, the content will remain the same for each group, but the frequency will differ. The intervention plan is shown in Table 1. All intervention content will be implemented through home self-monitoring and WeChat feedback. When patients experience suboptimal blood sugar monitoring, researchers will guide them in regulating self-management behavior. If behavioral regulation is ineffective, contact the attending physician for medication guidance. The outcome indicators will be collected at three time points: baseline, 12 and 24 weeks, respectively.

**Table 1:** Intervention plan of the the stratified support pattern-based internet-assisted self-management therapy for DM-MCI

Patient type	Intervention method	Specific content
High cognition, high management	1. Monitor fasting blood glucose or 2 h postprandial blood glucose 2–3 times a week and provide once a week reminders 2. Learn disease and health-related information and assess diet, exercise, medication, cognitive training, and mental health, once a week 3. Exercise 3 days a week for 20-–40 min each time and provide once a week reminders	**Dietary management:** Mediterranean cuisine, with Omega-3 as the core; Use more fruits and vegetables, fish, olive oil, nuts, beans, and coarse grains. Peanut oil, soybean oil, walnut oil and rapeseed oil can be selected as oil; Fish: Both marine and non marine fish are acceptable; It is not recommended to supplement Vitamins B, E, and complex vitamins to delay cognitive impairment. Recommend low GI foods to control or reduce weight **Exercise management:** 150 min of light to moderate aerobic exercise per week **Medication management:** Medication time, medication method, and medication contraindication **Blood glucose monitoring:** Oral hypoglycemic drugs are used to monitor fasting blood glucose or 2-h postprandial blood glucose 2–4 times a week. If blood glucose control is stable, the interval can be flexibly adjusted, and blood glucose measurement methods (Preprandial blood glucose, pre—post blood glucose matching) can be selected based on one's own blood glucose situation. Those who do not meet the standard for glycated hemoglobin should be tested every 3 months, and once every 6 months after reaching the standard **Cognitive guidance:** Knowledge of cognitive impairment diseases, prevention methods, influencing factors and so on. And appropriate reminders as cognitive stimulation. Cognitive monitoring is followed up every 12 weeks
High cognition, low management	1. Monitor fasting blood glucose or 2 h postprandial blood glucose 3 × a week and provide once a week reminders 2. Learn disease and health related knowledge and assess diet, exercise, medication, cognitive training, and mental health, twice a week 3. Exercise 3 days a week for 20–40 min and provide once a week reminders	
Low cognition, high management	1. Monitor fasting blood glucose or 2 h postprandial blood glucose 3 × a week and provide twice a week reminders 2. Learn disease and health-related knowledge and assess diet, exercise, medication, cognitive training, and mental health 3 × a week	
Low cognition, low management	1. Monitor fasting blood glucose or 2 h postprandial blood glucose 2–3 times a week and provide 3 × a week reminders 2. Provide correct disease-related knowledge and assess diet, exercise, medication, cognitive training, and mental health twice a week 3. Exercise 3 days a week for 20–40 min and provide 3 × a week reminders	
**Target range of blood glucose**	The **target of blood sugar control** is divided into three levels: strict, general, loose **The FBS is sequentially controlled as follows:** 4.4–6.1 mmol/L, 6.1–7.8 mmol/L, 7.8–10.0 mmol/L **The PBS is sequentially controlled as follows:**: 6.1–7.8 mmol/L,7.8–10.0 mmol/L,7.8–13.9 mmol/L	
**Requirements of blood sugar control**	① Strict control: Before and after elective or emergency fine surgery (such as plastic surgery) ② General control: People at high risk of cardiovascular and cerebrovascular diseases accompanied by unstable cardiovascular and cerebrovascular diseases, glucocorticoid therapy, selective large, small, and organ transplant surgeries, emergency organ transplant surgeries, surgical ICU patients ③ Loose control: People at high risk of hypoglycemia, hospitalized due to cardiovascular and cerebrovascular diseases, moderate to severe liver and kidney dysfunction, elderly people over 75 years old, life expectancy < 5 years (such as malignant tumors), mental or intellectual disabilities, before and after emergency large, small, and medium-sized surgeries, gastrointestinal or parenteral nutrition	
**Intervention implementation**	**Health knowledge learning and feedback:** □ Push disease related health knowledge in the form of official account □ Ask the patient questions related to the health knowledge pushed on that day □ Discuss key knowledge points in the group and invite patients to draw inferences from each other **Blood glucose monitoring, exercise, medication reminders:** Reminder in the form of WeChat chat box text **Interactive consultation:** Researchers establish a WeChat group where patients can discuss within the group. Participants can also send questions one-on-one to researchers for consultation	
**Intervention media**	WeChat	

#### Wechat operation

**Fig. 3:**
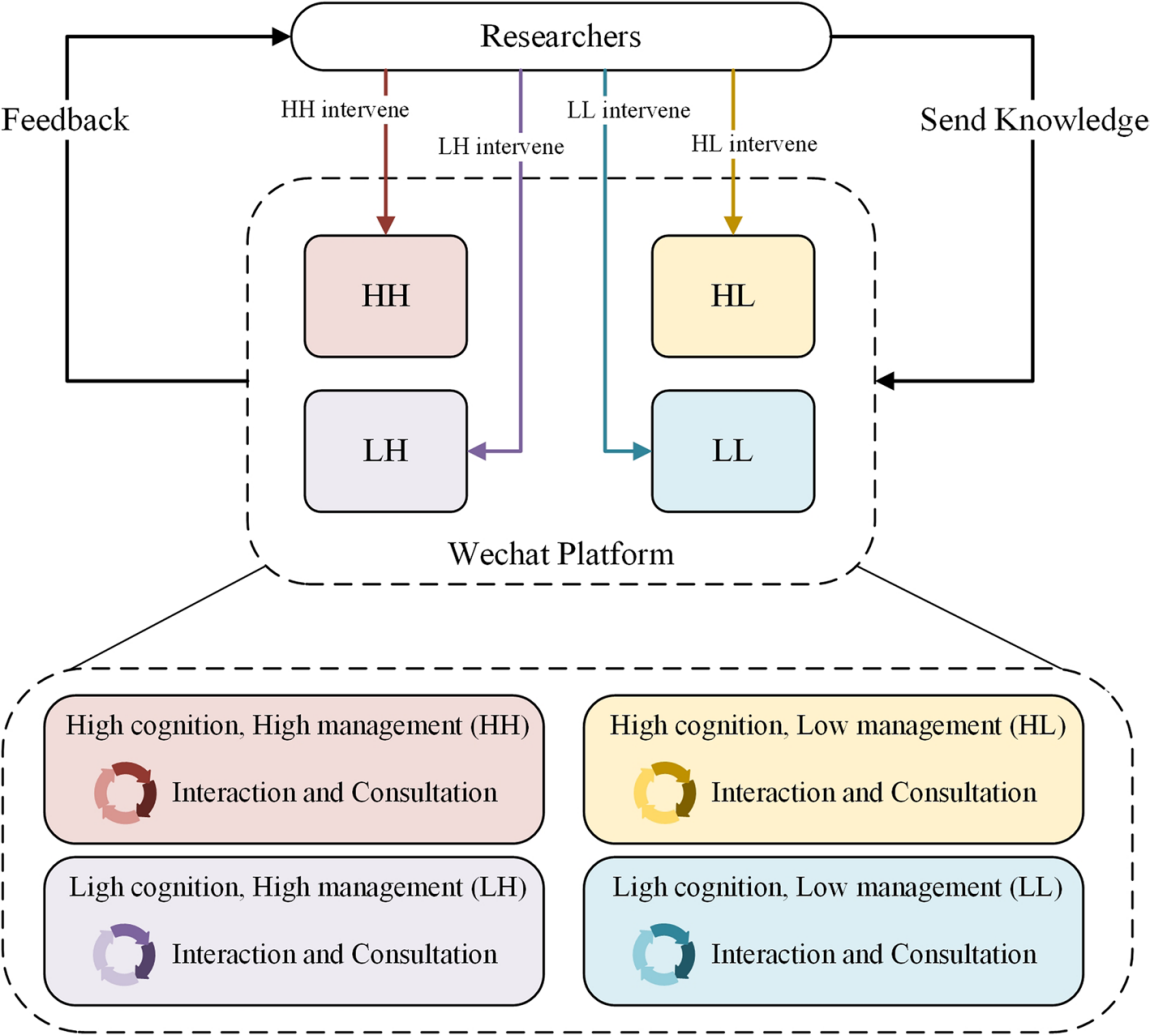
Flowchart of the Wechat operation for SISMT for DM-MCI

WeChat is the intervention medium for this study. The researchers will establish WeChat groups and created WeChat official account before the intervention. The researcher will send the completed content of the WeChat official account to the participants on the WeChat social platform. Participants can use the chat box function of the WeChat social platform to provide feedback and exchange on content of the WeChat official account, and can also interact with other participants in the WeChat group to share their self-management experience and personal insights on health knowledge. And they are required to use this application to inform researchers of their fasting blood glucose level, postprandial blood glucose level, hypoglycemia, diet and exercise status (Fig. 3).

### Control group

The control group will adopt the HMI. After the outpatient service, health education will be given to patients. We will provide patients with disease-related health manuals to help with the self-management of patients. The content of the health manual revolves around the epidemiological status, diagnostic methods, clinical manifestations, influencing factors, and intervention methods of cognitive impairment. Distribute a health manual and explain it to each participant in the control group. Outcome indicators will be collected at three time points: baseline, 12 and 24 weeks, respectively.

### Criteria for discontinuing or modifying

During the intervention period, if the participant is unwilling to continue due to personal reasons, the intervention will be terminated. Participants who are unable to continue but are willing to undergo follow-up will be modified for intervention measures and replaced with a control group.

### Adherence of intervention

Firstly, WeChat is adopted as the intervention medium and a multifunctional social media application in our study. Researchers can request participants to provide feedback and clock in on the intervention content to test the adherence of the intervention implementation. Secondly, Wechat official account has background data (the number of times and number of readers read), which can monitor the learning of participants. When the data does not match the number of participants, it will remind them in the Wechat group. Thirdly, establishing good cooperative relationships with participants fundamentally ensures adherence.

### Concomitant care

During the intervention process, participants should continue to take medication related to the disease. And inform patients before the intervention begins that they cannot participate in other intervention activities during the participation process. However, if participants participate in other interventions targeting outcome indicators such as blood glucose control and cognitive function during the participation process, their test data will be excluded.

### Outcomes

#### Outcome measures timeline

To compare the outcomes of the self-management interventions provided under the SISMT and HMI, a baseline (W0) survey will be administered to both the experimental and control groups before the start of each pattern. Outcome indicators will be collected 12 weeks (T1) and 24 weeks (T2) after the intervention to compare the effects of the two patterns (Table 2). The indicators will be assessed by experienced staff members who will be blinded to the group allocation. Outcome indicators and measurement tools are as follows (Domains /characteristics of tools see Table 3):

**Table 2:**
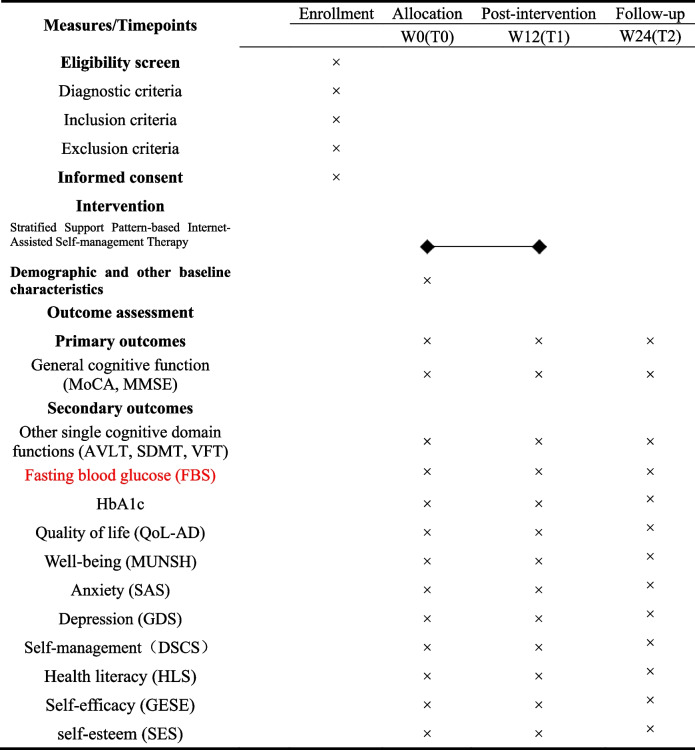
Time schedule of the a stratified support pattern-based internet-assisted self-management therapy for DM-MCI

**Table 3:** The domains/characteristics of tools

Tool	Domains/Characteristics
MoCA	Evaluate overall cognitive function; Developed by Nasreddine et al., it is an internationally recognized MCI screening tool that evaluates eight cognitive domains: attention, visual space and executive function, memory, language, abstract thinking, computation, and directional ability. It consists of 12 questions with a total score of 30 points. A score of 26 or higher indicates normal cognition, while a score of higher indicates better cognitive function. If the education period is less than 12 years, a score greater than 25 is considered normal, and the lower the score, the more severe the cognitive impairment of the patient; Detect Cronbach's α Coefficient 0.83, specificity 0.87
MMSE	Evaluate overall cognitive function; MMSE has a total score of 30 points, consisting of 30 items covering 7 cognitive domains: orientation, memory, attention and computation, language, and visual-spatial structure. This scale is used to evaluate the overall cognitive function of the study subjects and can effectively distinguish between normal individuals and elderly individuals with dementia. This study adopts the revised version by Wang Zhengyu et al. and sets an outlier threshold based on educational level. When the illiterate group scores ≤ 17 points, the primary school group scores ≤ 20 points, and the middle school and above group scores ≤ 24 points, it is considered abnormal
AVLT	This study adopts the Chinese version of AVLT developed by Guo Qihao and others from Shanghai Huashan Hospital, which has good reliability and validity and has important application value for clinical guidance and scientific research. The operation of this scale mainly involves the researcher reading 12 words: coat, driver, pants, crabapple, carpenter, lily, headscarf, wintersweet, soldier, magnolia, lawyer, and glove. After each reading, the patient is asked to repeat as many words as possible and record them. The number of repeated words in the 3 times is added as the instant memory quantity (i.e. N1 + N2 + N3); After 5 min, ask the patient to recall 12 words and say if they still remember them. The researcher recorded the number as short-term memory (N4); After 20 min, ask the patient about the number of words they remember as long-delayed memory (N5); Finally, a re-recognition test was conducted, with a total of 24 words and 50% of the vocabulary with 12 interfering items. The patient responded with "said" or "not said", and the researcher determined that each correct answer scored one point
SDMT	SDMT is commonly used for testing attention, learning, and conversion abilities. This test is a numerical symbol subtest in the revised Wechsler Adult Intelligence Revised in China (WAIS-RC), which reflects the information processing speed of the subject. During the testing process, participants were provided with a piece of paper with a template containing 9 digits, each with its corresponding symbol. The test requires patients to fill in as many and as quickly as possible the corresponding numbers of the symbols in the spaces below the numbers. The subjects should fill in as many and correct numbers as possible within 90 s. When it's time to calculate the correct number, the higher the score, the better the tester's attention and conversion ability
SAS	GDS is the SAS Self-Rating Anxiety Scale that was designed and developed by Zung in 1971 in the United States to measure the degree of individual anxiety symptoms. The scale has a total of 20 items, of which 5, 9, 13, 17, and 19 are scored in reverse. The scale adopts a scale of 1–4 (scoring method: 1 point for no or very little time; 2 points for a small portion of time; 3 points for a considerable amount of time; 4 points for the vast majority or all of time). Individuals fill out the scale based on their own situation in the most recent week, multiply the total score by 1.25, take an integer as the standard score, and use it as a statistical indicator. The higher the standard score, the more severe the anxiety level of the individual. The scale score can only serve as a reference indicator for evaluating anxiety and cannot be used for diagnosis
GDS	GDS is used to screen for depressive symptoms in the elderly population and is one of the most commonly used scales in China to evaluate the depressive status of the elderly. This study used GDS-15, which contains 15 items and can be used for rapid screening of elderly people with depressive symptoms. It is an internationally commonly used scale. Define a score of 0–4 as no depressive symptoms, 5–8 as mild depressive symptoms, 9–11 as moderate depressive symptoms, and 12–15 as severe depressive symptoms
MUNSH	This scale is used to measure the current level of happiness experienced by participants; Contains 24 items: each item is a description of emotions or experiences, requiring a response of "yes", "no", or "not knowing" based on recent life experiences; Regardless of whether the item is positive or negative, the answer "yes" is recorded as 2 points, "no" is recorded as 0 points, and "don't know" is recorded as 1 point. All items have a score range of 0 to 24 points, and the total subjective well-being score is equal to the positive item score minus the negative item score, with a value of -24 points to + 24 points. To avoid negative points, the total score is added to a constant of 24 points, and the final score range is 0 to 48 points
QoL-AD	This scale can be used to measure the quality of life of patients with cognitive impairment, reflecting the physiological, psychological, and social relationships, and some complex life abilities of the elderly. The scale consists of 4 dimensions and 13 subordinate items. The 4 dimensions are physical health and behavioral compliance, mental state, living environment and social relationships, and life satisfaction. Each item is scored into four levels, namely poor, average, good, and very good. The score is positively correlated with quality of life, with higher scores indicating higher quality of life. The different criteria are as follows: a QOL-AD score ≤ 26 points indicates a lower quality of life, a QoL-AD score of 27–38 points indicates a moderate quality of life, and a QoL-AD score ≥ 39 points indicates a higher quality of life. The scale Cronbach's α coefficient is 0.66; The intra-group correlation coefficient of the total score is 0.84
GSES	The Chinese version of the General Self-Efficacy Scale was first used in 1995, and some studies have also used GSES to measure self-efficacy in individuals with mild cognitive impairment. The scale consists of 10 items, using the Likert 4-level scoring method. The lowest score is 10 points, and the highest score is 40 points. The higher the score, the better the self-efficacy, that is, the stronger the confidence in dealing with things. The scale has good reliability and validity, Cronbach's α The coefficient ranges from 0.76 to 0.90
SES	This scale was developed by American psychologist Rosenberg in 1965 and has good reliability and validity. This study used the Chinese version of the self-esteem scale revised by Ji Yifu et al., consisting of 10 items, to measure the overall self-esteem level of a single dimension through self-evaluation. Using a four-level scoring system of 1 to 4 points, representing very non-compliant, non compliant, compliant, and very compliant. 1. 2, 4, 6, and 7 are forward counting items, while 3, 5, 8, 9, and 10 are reverse counting items. The total score range is 10 to 40 points, and the higher the total score, the higher the level of self-esteem
HLS	The scale is based on Nutbeam's hierarchical model of health literacy as the theoretical framework to establish an item pool that includes three dimensions: interactive health literacy, critical health literacy, and functional health literacy. There are a total of 19 entries. The content validity index of the total scale is 0.972, and the content validity of each item is 0.83–1.00. Cronbach's α The coefficient is 0.945, with Cronbach's for each dimension α The coefficients are 0 894, 0.909, 0.877. The scale adopts the Likert 5-level scoring method, with scores ranging from "cannot" to "completely possible" ranging from 1–5 points. The higher the total score, the better the level of health literacy
DSCS	Assess the self-management ability of diabetes patients; This scale was developed in 1992 by Hurley and Shea and was simplified into Chinese by domestic scholars Wang Jingxuan et al. Cronbach's α coefficient is 0.92. The scale consists of six dimensions: dietary management, blood glucose monitoring, foot care, exercise management, medication management, prevention, and management of high and low blood sugar. There are a total of 26 items, with a Likert score of 5 points. A score of 1–5 points corresponds to a total score of 26–130. This scale has good reliability and validity when used in both Taiwan and mainland China

#### Primary outcomes

The primary outcome measures will be general cognitive function (MoCA [35], MMSE [36]).

#### Secondary outcomes

The secondary outcome measures will include several commonly used measures of specific domains of cognitive function, psychological indicators, blood glucose control (FBS, HbA1c, PBS) and other relevant indicators. The measures of specific domains of cognitive function (memory, language, attentions) will comprise the Auditory Verbal Learning Test (AVLT) [43], Symbol Digital Modalities Test (SDMT) [44]. Psychological status will serve as the secondary index of this study. This will include anxiety, depression, well-being, quality of life, self-efficacy, and self-esteem. The Self-Rating Anxiety Scale (SAS) will be used to measure anxiety [45] and the Geriatric Depression Scale (GDS) will measure depression [46]. Well-being will be assessed using the Memorial University of Newfoundland Scale of Happiness (MUNSH) [47]. Quality of Life-Alzheimer’s Disease (QoL-AD) will be used to assess patient quality of life [48], the General Self-efficacy Scale (GSES) will be used to measure self-efficacy [49], and the Self-Esteem Scale (SES) will be used to measure self-esteem [50]. Other relevant indicators include the healthy literacy and self-management behavior which can be used to investigate the patient’s acceptance of the intervention content. Health literacy and self-management behavior will be determined using the Health Literacy Scale for type 2 diabetes and the diabetes self-care scale (DSCS).

### Data collection

FBS, HbA1c, and PBS will be measured and collected from participants' self-reported data. Other data that need to be measured using a scale will be invited participants to the hospital again for measurement and collection.

### Statistical analysis

All data analysis will be performed using SPSS Statistics v21.0 (IBM). To ensure that they are blinded to group assignment, the outcome assessors and data analysts will not interact with the participants except during data collection (in the case of outcome assessors). Demographic and other baseline characteristics will be summarized using descriptive statistics. If necessary, the results will be adjusted for potential confounders, such as age, gender, and education. Quantitative data will be expressed as frequencies or rates and statistical analysis will be carried out using the *χ*^2^ test. If these data are normally distributed, they will be expressed as (*x* ± *s*). The effects of the interventions (between-group differences) will be calculated with linear mixed models using interaction terms (group allocation versus time), which are equivalent to the between-group differences. The within-group differences will be calculated using repeated measures ANOVA. *P*-values < 0.05 will be considered statistically significant. The data will be reviewed before analysis. The Statistical Package for the Social Sciences (SPSS) software will be used for all analyses. The clinical relevance of the results will be confirmed by calculating the effect size (Cohen d) of the significant differences found between the assessments. The following effects will be considered: small: 0.00–0.49; medium: 0.50–0.79; high ≥0.80 (Cohen, 1988). Comparison between the four subgroups within the intervention group using ANOVA test.

## Discussion

The purpose of this study is to determine whether the related intervention research on a SISMT can improve the self-management behavior of patients with DM-MCI and thus reduce disease symptoms and increase quality of life. Self-management is very instructive for patients with chronic diseases. This is especially true for older adults with chronic diseases who are highly susceptible to negative outcomes [51]. Thus, the self-management behavior of older patients with DM-MCI is of particular concern. However, there is great heterogeneity in both these diseases and the patients they afflict [52, 53]. The stratified support model is a great way to improve the efficiency of resource utilization, not only saving human resources, but also satisfying patient needs to a greater degree. A prior study used the triangle stratification model to manage diabetes patients, and primarily stratified by patient age, glycosylated hemoglobin, blood glucose, and other objective disease characteristics [54]. By considering both patient characteristics and demands, including cognitive function and diabetes self-management as sub-variables, we can construct a subgroup of patients with DM-MCI, and implement self-management interventions based on subgroup characteristics. Patients may receive knowledge that is not conducive to disease recovery, management burnout, and other situations during long-term self-management [55]. Dynamic monitoring can play both a reminder and a supervisory role, improving the efficiency of self-management and better controlling diseases. The internet is likely to meet this demand [56]. As a new element of social connection, it can partially replace connections between patients, medical care practitioners, and society at the practical level, strengthening self-management, realizing the maximum reconstruction of social connections, and promoting cooperation and interaction between patients and all parts of society [57].

### Limitations

This study has some limitations. First, the design does not allow blinding of the intervention implementer. Second, the effect of a long-term intervention will remain unknown following study completion. Third, there may be a selection bias in the participant pool given the requirement for patients to use smartphones independently. In addition, since participants in the intervention group may tend to provide a more positive response, the self-reported questionnaire may introduce a potential bias and affect the accuracy of the trial to some extent.

## Conclusion

This study adopts a SISMT to strengthen patient understanding of self-management behavior-related knowledge in a more personalized way, improving self-management ability, increasing blood glucose control, and reducing cognitive dysfunction. The research results will provide evidence to related intervention research on the SISMT.

## Data Availability

The datasets generated during and/or analyzed during the present study will be available from the corresponding author on reasonable request.
